# Parallelization: the Fourth Leg of Cultural Globalization Theory

**DOI:** 10.1007/s12124-021-09600-4

**Published:** 2021-01-20

**Authors:** Björn Boman

**Affiliations:** Department of Education, Stockholm University, Frescativägen 54, 106 91 Stockholm, Sweden

**Keywords:** Hybridization, Global culture, Polarization, Homogenization, Climate change, Capitalism, Parallelization

## Abstract

Extending Pieterse’s (1996) tripartite cultural globalization theory consisting of homogenization, hybridization and polarization, the current article outlines a set of exemplifications and justifications of a fourth theoretical underpinning labeled parallelization. The theory implies that at a global scale, crucial events that appear paradoxical or contradictory occur at the same time, such as carbon emissions due to growth-fixated global capitalism, while the causes of carbon emissions lead to greater resilience against the consequences of carbon emissions as wealth accumulates. Other examples discussed are large-scale migration flows which lead to increased segregation in host societies while integration of migrants occur as a parallel process; secularization visa-à-vis the resurgence of religions; clear indications of that the biological component of cognitive abilities decreases due to fertility patterns in many locations around the globe, while the IQ test scores have risen as a consequence of various environmental factors.

## Introduction

At this point, a rich literature on cultural globalization theory exists (e.g., Pieterse 2015; Pieterse 1996; Pieterse 1994; see also Harvey 2007). There is also a plethora of crucial works on the nexus between globalization and political-economic policies (e.g., Rodrik 2011), as well as globalization, religion, and secularization (e.g., Casanova 2007; Gorski et al. 2012; Norris and Inglehart 2012; Berger 1999; Riesebrodt 2010). Even cognitive abilities have been analyzed on a global scale (e.g., Flynn 2012; Rindermann 2018). Many journals in sociology, communication studies, and psychology are, or have been, deeply engaged in these critical discussions for several decades and other scholars can now easily attach their works to well-established conceptual frameworks that conform to their ideas and empirical data. For instance, researchers in cultural studies can analyze cultural phenomena, at the local, regional, cross-country, and global level, by means of useful concepts such as homogenization, hybridization, and polarization.

Homogenization was constructed via the term McDonaldization, elaborated by Ritzer (1993), while scholars such as Hannerz (1992), Fairclough (1992), Bhabha (1994), and Greenfeld (1994) have established the conceptual nodes associated with hybridization, such as transnationality, cultural hybridity, and intertextuality. Laclau and Mouffe (2001) and Huntington (1993, 1996) have enhanced the understanding of polarization (although seldom using the term specifically) by highlighting various dimensions of cultures (Huntington) and politics (Laclau and Mouffe) as tectonic plates that lead to friction or antagonism. These have then been discussed as a tripartite cultural globalization model by Pieterse (1994, 1996, 2015), who generally emphasizes globalization as a manifestation of hybridization (Pieterse 1994; Pieterse 2015).

However, there is another theoretical underpinning which needs to be further addressed since it transcends the triad elaborated by Pieterse and others, and which highlights processes that go beyond the earlier concepts but is of, at least, equal magnitude and significance. I suggest it to be labelled *parallelization*, in science used to signify a certain meaning in applied mathematics and computation (e.g., Schieber and Vishkin 1988) but with no such mechanistic meaning in the social sciences, because it is indeed the case that many related events and processes are taking place at the same time, and which give rise to paradoxes (e.g., the secular rise of intelligence, Lynn 1996) since such processes are hard to discern and transcend regular unilinear ways of thinking and reasoning. One might identify this, for instance, in relation to climate change (and discourses on climate change), socioeconomic and sociocultural integration of migrants in host societies, and secularization visa-à-vis the resurgence of religion, as well as global cognitive ability patterns. For example, there is growing body of evidence that suggests that massive economic growth directly or indirectly has led to climate change through carbon emissions, and that economic growth leads to greater resilience to the consequences of climate change and perhaps unrelated cataclysms (Nordhaus 2015; Galiana 2018). Furthermore, cognitive abilities rise (through environmental input) and decline (through socioeconomic fertility patterns) in parallel processes when lower-SES cohorts of individuals procreate and at the same time gain greater access to education, nutrition, and modern infrastructure.

The current article aims to describe four instances of how parallelization takes place on a global scale and might be seen as an important complement to homogenization, hybridization, and polarization (as well as other complex cultural models) and as a manifestation of cultural, biological, and psychological complexity as it is related to both concrete physical processes and the way these are considered.

It proceeds with an overview of Pieterse’s tripartite model with some additive critical remarks. Then the theoretical concept of parallelization is fleshed out in relation to the four instances briefly mentioned above. The intention is not to analyze these complex phenomena in depth, but they are all important and fascinating instances which illustrate the author’s argument. The analytical descriptions rest on the assumption that these are global phenomena but must often be understood locally. It ends with a conclusion and suggestions for future research.

## The Tripartite Cultural Globalization Models

As said above, Jan Nederveen Pieterse (1996) has outlined three major cultural tendencies in the age of globalization: homogenization, hybridization, and polarization. Homogenization implies a westernized, particularly Americanized, imposition of culture and consumer products on virtually all societies which are reached by its neoliberal agenda (Pieterse 1996; Harvey 2007), while hybridization signifies the blend of local and global practices or local and other local elements (Pieterse 1994). Hybridization is partly associated with postcolonial culture theory (Bhabha 1994) and is more concretely related to for instance syncretism of religions and merging of languages (creolization) (Pieterse 2015), populational mixing (Cavalli-Sforza 2001; see also Moghaddam 2009), and a substantial part of modern and contemporary popular culture (Pieterse 2015). This viewpoint also criticizes the Eurocentric perspective which accentuates globalization as westernization, a process that supposedly started in the sixteenth century or later. Rather, hybridization takes the concept of world cultures as a point of departure. The West “itself” is not seen as monolithic (Pieterse 2015).

For example, the now successful South Korean music genre K-pop might be characterized as a combination of Korean traits (e.g., Korean language, predominantly Korean-born artists, companies located in Seoul) and Western components (e.g., capitalism, consumerism, and fashionable pop music genres, typically originating from the US, and roughly 20% of English vocabulary). K-pop is thus an example of both hybridization and homogenization, because despite the local/global fusion (i.e., hybridization) it majorly accepts the dominance of American capitalist culture (Lie 2014; Jin and Ryoo 2014; Yoon 2017).

Polarization, on the other hand, is connected to Huntington’s (1993) notion on the emergence of global conflicts between some of the world’s nine major civilizations, majorly the Islamic world versus the West and the “Sinic assertiveness” to reach regional if not global hegemony (Huntington 1996). Ferguson (2011) has noted that since the book was released, indeed most conflicts have occurred within, not between civilizations. For example, the regional hegemonies of the Islamic civilization – Iran and Saudi Arabia – battle each other more than the US. Saudi Arabia and the US have political-economic strategic alliances. Therefore, Huntington (1993) downplays the political and economic dimensions while giving precedence to religious, cultural, and ethnic markers, at the expense of analyzing international relations correctly.

Another instance of the co-existence of polarization and hybridization is the geopolitical conflicts and sociocultural markers in the Donbas region in eastern Ukraine (e.g., Gentile 2015). Huntington (1996) stresses that Ukraine is a torn country, divided between the West (EU/Kiev) and East (Russian-speaking minorities/Russian Federation). The armed conflicts in eastern Ukraine 2014 onwards give support to a notion of latent conflict related to ethnolinguistic, economic, and geopolitical preferences (Huntington 1993). It does also, in part, corroborate Ferguson’s (2011) insight about the predominance of inter-civilizational conflict during recent decades. According to Huntington (1996), Ukraine does partially belong to the Orthodox civilization while Russia is this civilization’s core country. The polarization between Ukrainian forces and pro-Russian separatist forces within Ukraine is indisputable, whereas the ethno-linguistic and ethno-cultural hybridization is less palpable. Typically, however, people must speak Russian within the self-proclaimed Luhansk People’s Republic, as most already did prior to the armed conflict in 2014. However, those who identify as Ukrainian *and* prefer the Ukrainian language must either artificially switch between Ukrainian and Russian or mix the two to some degree and then with the risk of some social stigmatization for maintaining a Ukrainian accent and vocabulary (e.g., Gentile 2015).

As Gamsakhurdia (2020; see also Gamsakhurdia, 2019) notices, a general civilization theory implies an overly simplistic understanding of states, people, and culture at the macro level which stresses the unreal isolation of such subjects and geographical locations. In reality, no such civilizations exist. This is perhaps why Pieterse (e.g., Pieterse 1996; Pieterse 2015) emphasizes the hybrid reality and potentiality that signifies the world, both historically and currently. Moreover, it might imply the shortcoming of Pieterse’s tripartite model as these different tendencies may not be as distinct as it seems at first glance. Nevertheless, the distinctive feature of polarization is cultural conflict (Pieterse 1996; Huntington 1993).

The distinction between weaponized antagonism and principally verbal disputes is the reason why influential scholars within political science, most notably Chantal Mouffe (e.g., Mouffe 1999; Mouffe 2005; Mouffe 2013), have re-elaborated and reassessed the German professor of law, Carl Schmitt’s concept of political friends and enemies (Schmitt 2009) related to pluralism within democratic societies and tense international relations. Instead of enmity it is more suitable to underline the existence of so-called *agonism*, i.e. antagonism which is not associated with physical conflict but by discursive divergence or dissensus.

While agonism is mainly related to democratic politics and a critique of Habermasian liberal democracy’s insistence of consensus, of which the latter aims to reduce politics to economic cooperation and the aim of harmony among a plurality of parties (Mouffe 2013; Mouffe 1999; Mouffe 2005), it is applicable to international relations and non-political contexts, both in public and private. Mouffe (2013) argues, partially along the lines of Schmitt (2009), that since the world constitutes a pluriverse and society is driven by human passions and contingency – things could be different, objects and subjects are never completely fixated – there is always a risk of emerging agonism (Mouffe 2013).

In the present global world, it is relatively easy to pinpoint instances of all these tendencies, both within and between various localities. For instance, cultural particularities in both Japan and South Korea are merged with Western elements such as capitalism, consumerism, and liberal democracy – signifying both homogenization and hybridization – and with regard to their international relations there are instances of agonism linked to the colonial past (e.g., Jonsson 2015; Lie 2014; Inoue 2007). Perhaps another partial disadvantage of Pieterse’s tripartite model is that homogenization and hybridization do only constitute differences of degree because homogenization is hybridization with dominant western features. Nonetheless, the tripartite cultural globalization model is pertinent and still significant to describe tendencies, although simplified. However, it does not account for other forms of processes which do not fit neatly into any of the other three frameworks and that are rather linked to parallelization.

Moreover, it does not elaborate on the basic definition of what culture means without these conceptual abstractions, perhaps because many social scientists act elusively in relation to this pervasive concept (Gamsakhurdia 2020). Whereas the current article does not lay out even a preliminary working definition of the term “culture” to have as a point of departure, as it is difficult to find a common definition (Jahoda 2012), it is the author’s intent to overcome many of the dichotomies such as relativism–universalism and offer a more complex and encompassing understanding that views internal and external (or physical) processes as complementary rather than binary (e.g., see Valsiner 2014; Gamsakhurdia 2020). For instance, climate change is a physical and external process but individuals’ and groups’ understanding of it is, indeed, related to semiotic and internal systems and interpretations. This might extend to other phenomena being discussed, such as secularization, economic growth, and cognitive ability.

## Parallelization

Below the four exemplifications and illustrations of parallelization are examined. These parallel processes, related to a particular main phenomenon, are also listed in Table 1 to gain a clearer picture of the argument.

**Table 1:** Parallelization: the fourth leg of cultural globalization theory

**Global phenomena A-B: Economic growth and climate change** Pattern 1: Economic growth leads to carbon emissions Pattern 2: Economic growth leads to greater resilience against natural disasters and future consequences related to carbon emissions, such as rising sea levels	
**Global phenomenon C: Migration flows** Pattern 1: Increased segregation and inequality due to large inflows of low-skilled migrants Pattern 2: Increased integration and socioeconomic inequality of migrant groups	
**Global phenomena D-E: Secularization and religious resurgence** Pattern 1: Increased segregation and inequality due to large inflows of low-skilled migrants to countries like Sweden, US, UK and France Pattern 2: Increased integration and socioeconomic inequality of migrant groups	
**Global phenomenon F: IQ** Pattern 1: The genetic dimension of IQ deteriorates slowly in many countries due to fertility patterns Pattern 2: Environmental factors cause the measured IQ to rise in most locations, i.e. the Flynn effect takes the upper hand (but might come in reverse)	

### Economic Growth and Climate Change

There is a strong consensus about that anthropogenic climate change has occurred and that the planet is increasingly warming. Therefore, social scientists are more interested in how to respond to these issues from for instance a cost/benefit analysis (CBA) perspective rather than to deny that they exist (e.g., Galiana 2018; Nordhaus 2015; Cai and Lontzek 2019). Economic growth, on a regional and global scale, has led to climate change due to massive carbon emissions. This is one major process that has occurred since the nineteenth century, with an acceleration throughout the post-World War II era. In 2007, China became the world’s largest emitter after the US (Zhang 2011).

On the other hand, economic growth does also lead to greater resilience against the effects of the warming that has occurred (and many that may not be related to climate change but to natural variability, like earthquakes), such as a robust infrastructure (Galiana 2018; Clarke et al. 2009; Zhang 2011), which has escaped the attention of scholars such as Geels (2014) and Schwartz (2019) who only emphasize the negative effects of global capitalism. For example, when a hurricane hits Florida it does only cost a small micro-percentage of the total aggregated GDP of the state, as well as the federal GDP. However, if a similar event occurs in Guatemala or the Philippines it is much costlier and damaging, both in terms of economic costs and human lives. While perhaps not related to global warming, the earthquake that affected Fukushima Daiichi Nuclear Power Plant (NPP) in 2011 did not have much of a large-scale effect as a consequence of Japan’s strong facilities and general resilience against natural disasters. “Only” about 2000 people died in the aftermath of the post-cataclysmic evacuation. This can hardly be said about South Asia and Southeast Asia, which were deeply affected by the tsunami that hit 14 countries and two major regions in 2004 and killed approximately 300,000 people and left millions of people homeless. The tsunami in 2004 was larger and more widespread compared to the earthquake near Japan’s east coast (Yasumura 2014; see also Symaco 2013, in relation to the Philippines), but the point is, yet again, to stress the significance of preparation, resilience, and appropriate well-developed infrastructure (for critical perspectives, see Schwartz 2019; Morselli 2013). Economic growth is, at least at some point in history, like an armed robber who shoots a victim and then pays the hospital bill, in particular for the wealthy.

However, this is not really a “dialectical” process but rather two parallel processes that occur more or less simultaneously. They can be interrelated or interdependent, since capitalism, as related to economic growth, is used to address the problems that it in part has caused, but they are also distinct trajectories. Such issues need to be taken into consideration when discussing complex issues like climate change and its discontents, which often tend to lead to dichotomous discursive positions about either a suggested “business as usual model” with some adaption and emissions cuts through technology, typically propagated by capitalists (Nordhaus 2015; Zhang 2011; see also Cai and Lontzek 2019), or anti-growth campaigns that go in the opposite direction.

### Migration Flows

Another instance of parallelization is the patterns related to integration of migrants in various national and local contexts. For example, economists such as Sanandaji (2018) and migration scholars like Vogiazides and Mondani (2020) have argued that the socioeconomic and sociocultural integration of migrants in Sweden has failed, especially in the city of Malmö, leading to increased fiscal burden, high unemployment rates and crime rates (Sanandaji 2018). The Swedish society is also becoming increasingly more segregated according to Sanandaji (Sanandaji 2018, see also Ekberg 1999). The discourse of relative failure of the implementation of migration and integration policies has become more frequent and concerted with the rise of the nationalist and right-wing populist party the Sweden Democrats (SD), which has become the country’s largest political party according to some recent polls (e.g., Nyheter idag 2019).

However, this viewpoint does not account for the parallel integration that occurs simultaneously as the migration, which, on the other hand often leads to increased segregation and socioeconomic inequality. For instance, in the 1970s and 1980s, people from Finland, Greece, Yugoslavia, Chile, and Iran were looked upon as the “other”. Now the broader host society considers them quite prototypical Swedes and often with better-than-average levels of educational attainment (Gärdqvist 2006). Indeed, this is an exemplification of successful socioeconomic and sociocultural integration. Instead, Swedes with a xenophobic or anti-immigration agenda have moved on to target other groups, such as beggars from Romania and Bulgaria (Malmqvist 2015) or emphasize the sheer numbers of low-skilled migrants as the major issue (Sanandaji 2018).

The binary between those that argue that migration leads to segregation (i.e., the migration and integration has failed discourse) must consider the parallel process of integration that has succeeded. The other way around is also true. While the Swedish context is an example briefly used to highlight this issue, it might be equally appropriate to apply the concept of parallelization to for instance the US, UK or France which have faced somewhat similar patterns of migration flows and socioeconomic processes. For instance, prior to the election of Donald Trump, as well as afterwards, Mexican Americans have been targeted as an unsuccessful migrant group relative to whites and Asians. However, as Lee and Zhu (2015) explain, a substantial share of this group has outperformed their parents in terms of educational attainment and earnings. This does, indeed, go against the discourse of segregation and failure. While not disregarding the problems associated with migration, inequality and segregation in the United States, California in particular, the example signifies that two major processes are occurring in parallel.

### Secularization and the Resurgence of Religion

The gradual rationalization and secularization of the world thesis stems from Max Weber and later accounts such as Martin (1978), Casanova (1994), Norris and Inglehart (2012), and Martin (2005), and essentially points to correlations, if not casual relationships, between scientific and economic development and decreased religiosity (i.e., secularization). In addition to describing, explaining, and predicting further secularization, the theory has implied that religious pluralism increases when the state promotes secularism instead of state-sanctioned religion, i.e., the split between church and state, but that nominal religious adherents become less pious and serious about their commitments as the society becomes wealthier and more modern (Martin 2005). Further, the thesis does, ad hoc, explain other complex or seemingly contradictory patterns such as increasing numbers of Christians and Muslims by for instance increased birth rates in poor Christian and Muslim countries (Norris and Inglehart 2012), while others are skeptical about the theory’s general validity (e.g., Stark 1999; Berger 1999; Riesebrodt 2010; see also Heelas 1996). Berger (1999) and Stark (1999) mention the US, Russia, South Korea, and Saudi Arabia as examples of countries which have experienced modernization and massive economic growth while remaining a strong sense of religious participation beyond mere nominal adherence.

Yet others have emphasized the necessity of a more multifaceted theoretical framework that accounts for the historical records and complex empirical realities around the world in relation to religious resurgence and secularization (Gorski and Ates 2008; Gorski et al. 2012). For instance, Gorski et al. (2012, pp. 6–7) stress that secularization theory, as laid out by Casanova (2007), consists of three core assumptions and underpinnings: 1) decline of religious belief, 2) the separation of religious and non-religious spheres, 3) the privatization of religious commitments. They reject number one and three but consider the second to be largely true, especially in Western countries. Riesebrodt (2014) explains how secularization theory went too far in its analytical scope and conclusions:

Differentiation produces relatively autonomous social spheres and frees them from religious control. This applies in particular to the separation of church and state, but also leads to the emergence of various institutional orders, like the economy, politics, and secular culture, which now can pursue their own goals and develop their own rules without being constrained by religion. But, obviously, this process of differentiation also institutionalizes religion as a separate social sphere. It was this displacement of religion from a force permeating society as a whole to a sphere of its own that originally has been understood to be the central feature of secularization and was believed to be an undisputed necessity for the emergence of truly modern societies. Had secularization theorists stuck solely to the thesis of institutional differentiation, the secularization debate would have been less confusing. But, unfortunately, most scholars made the concept of secularization much more complex. Many reasoned that this institutional differentiation should imply a general decline of religion. Religion would be relegated from the center of society to the periphery; science would replace religious beliefs; religion would disappear from the public sphere and become primarily a private matter; religious associations and participation in religious ritual practices would decrease.

Further:

Given these strong opinions about the role of religion in the modern world, hardly anybody was prepared for the dramatic resurgence of religion that happened since the late 1970s. Just to remind you of some events:

- in 1979 we witnessed the Islamic Revolution in Iran and the war of the Islamic mujahidin against the Soviet occupation of Afghanistan.
- in 1980 Ronald Reagan was elected president of the United States with the support of politicized evangelical Christians, but also Catholics, Jews, and Mormons.
- in Israel religious nationalists challenged secular Zionism.
- in Palestine the first Intifada shifted power from secular nationalists to Islamist groups.
- in India Sikh separatists challenged the secular state; and after the violent conquest of the Golden temple in Amritsar, Sikh bodyguards assassinated Indira Gandhi. The 1980s also saw the rise of the Hindu-nationalist BJP party.
- in Poland Solidarnosc with support from the Catholic Church challenged the Communist state.

With regard to Pieterse’s (1996) tripartite model there is some evidence for both homogenization (i.e., the diffusion and acceptance of Westernized secularization or atheism), hybridization as religious syncretism (e.g., Baker 2008; Pieterse 1994), and polarization (for a critical discussion of polarization between religious adherents and atheists, see Ribberink et al. 2018). Pieterse (2015) focuses on syncretic religions of the past and present, while Huntington (1996) emphasizes for instance the clashes between especially Muslims and Christian or atheist Westerners in the Balkans in the early 1990s. Ferguson (2011) mentions Shia and Sunni clashes as a more pervasive pattern in the Muslim world than intercivilizational conflicts, while Inoue (2007) sheds light upon Japan’s imperialist past in relation to China and Korea. Baker (2011) has noted some degree of polarization between Korean Christian intrusive missionaries and Muslims. Alesina et al. (2003) have linked religion to different degrees of fractionalization around the world, which is a conceptualization similar to polarization. However, in many of these instances it is difficult to discern to which extent these issues are political-historical rather than religious, or perhaps both.

Nevertheless, with regard to secularization and the resurgence of religion, it has become rather apparent that secularization and a strong presence of religion – such as in the US, Russia, France, UK, and South Korea – are simultaneous processes (Riesebrodt 2010; Gorski et al. 2012). Sweden is another interesting example, since the number of mosques has increased while the bulk of the host society is the most secular-individualistic in the entire world (World Values Survey 2019; see also Harlow et al. 2013). This may lead to a form of secular individualism intimately intertwined with New Age spirituality such as yoga, veganism, and “Oriental” influences (Heelas 1996) quite predominant in contemporary Sweden. Riesebrodt’s (2010) stricter definition of religion may exclude such elements but it nevertheless demonstrates additional complexity in regard to the secularization debate. On the other hand, Riesebrodt (2014) explains the parallel processes as following:

However, scientific thought has by no means replaced religious thought, disenchantment has been always accompanied by re-enchantment. There exist many industries, which profit from the reenchantment of the world. Hollywood and Bollywood produce modern myths, stores offer a huge selection of books on spirituality, esotericism, mysticism, and new age, and many Internet sites are specialized on do-it-yourself religions. Obviously, disenchantment and re-enchantment take place simultaneously. For many people they do not seem to compete with each other. They believe in medicine but also bring offerings to a temple and pray.

Malaysia, and various locations in the Gulf region such as Qatar and Dubai, is another fascinating instance of religious/secular parallelization. On the one hand, Islam has become fortified as a hegemonic religion in Malaysia. Tong and Turner (2008) stress the resurgence of popular Islamic religion in this country. This pattern is related to lower fertility rates among Malaysian women, which somewhat paradoxically leads to greater possibility of religious activities for “pious” Muslim females. In that sense there is some interdependence between secularization (lower birth rates signify the transition into a secular society, see Norris and Inglehart 2012) and “de-secularization”, but on the other hand, non-Muslims, such as the Chinese and Indian minorities, are not required to conform to the same strict sharia laws as the Malay-Muslim majority (Moustafa 2014). In tandem with secularized tourists they can easily get inebriated at a nightclub in Kuala Lumpur, albeit with some imposed alcohol tariffs on top of the bar tab (e.g., https://theculturetrip.com/asia/malaysia/articles/the-10-best-nightclubs-in-kuala-lumpur-malaysia/). This shows that in this local context the patterns are parallel, although not without friction due to Islamization in recent decades (Tong and Turner 2008).

Instead of arguing for the predominance of one particular pattern (secularization *or* the resurgence of religions), at least on a global scale, one may account for the parallelization of these two macro processes with a whole set of intertwined micro processes that give precedence for any of the two modalities.

### IQ: The genotypic decline of intelligence and Flynn effect.

Research on measured cognitive abilities (IQ) may seem controversial and contentious and sometimes it is, such as to which extent individuals from different social strata are less intelligent in a neurobiological sense and how the interactive genetics–environment constitution plays out in real societies (Nisbett et al. 2012; Haier 2017; Flynn 2012; Plomin 2018; Ceci and Williams 2013). However, the nexus between cognitive ability and class simply means that in a statistical sense it is more likely that for instance among the British working class the IQ, which is of at least 40% associated with genes (Nisbett 2010; Turkheimer et al. 2003; Engelhardt et al. 2018), is lower than among the upper-middle class. Since the lower-SES cohorts have more children than the higher-SES cohorts, on average, this has led to a slight decline in the genetic selection for higher cognitive ability levels, starting around the mid-nineteenth century when living conditions and medicine improved (Lynn 1996). Furthermore, this seems to have emerged as a global pattern (Lynn and Harvey 2008), although in Sweden an opposite pattern has been identified (Kolk and Barclay 2019) and thus refuting some of Lynn (1996) and Lynn and Harvey’s (2008) research. Nonetheless, Lynn and Harvey (2008, p. 113) assumes that the genotypic dimension of IQ has declined by 1.22 points from 1950 to 2000.

At the same time, another parallel process related to cognitive ability is occurring, that of the well-documented rise of measured IQ scores, known as the Flynn effect after the American-New Zealander political scientist James Flynn, due to various components of environmental input (e.g., Flynn 2012). Since IQ is affected by environmental factors like educational quality and quantity (Ritchie and Tucker-Drob 2018; Ceci 1991), nutrition (Lynn 2009; Nisbett 2010), stress and mental health (Nisbett 2010), and various aspects of the test-taking situations, such as motivation (Borghans et al. 2011) and increased proclivity to guess in regard to multiple-choice tasks and items (Pietschnig and Voracek 2015; Rindermann 2018), and perhaps the modern society’s focus on scientific spectacles and visualization (Flynn 2012), the rise of IQ scores throughout much of the twentieth century comes as no surprise. As soon as developing countries gain access to more modern education, infrastructure and lifestyles, the scores increase, which indeed have been the case in for instance South Korea, Saudi Arabi, Kenya, and Qatar during the late twentieth century and beyond (Flynn 2012). On the other hand, an apparent reason for a slight decline in cognitive test scores due to environmental factors seems to be that, young males in particular, read less as a consequence of the preference for TV consumption over books (Hernæs et al. 2019). Returning to Lynn (1996), the paradox is explained as follows:

There is an abundance of evidence that in the economically developed nations during the twentieth century, people with higher intelligence had fewer children than those with lower intelligence. The effect should have been that the level of the intelligence of the populations declined in the middle decades of the century. This effect was confidently predicted by people like Cattell (1937), Burt (1946) and Thomson (1949). Yet the predicted decline did not occur. On the contrary, the level of intelligence of the populations of the economically developed nations increased substantially during the middle decades of the twentieth century. How can this paradox be resolved? (…) It is quite possible for the phenotypic intelligence of the population to show an increase while the genotypic quality is in decline. To take an agricultural parallel, it is possible to sow seeds of deteriorating genetic quality in successive years and to pump in so much more fertilizer that the size of the crop actually improves. This is probably what has happened with intelligence, although there is no way of making a direct test of the theory that the genotypic intelligence of the population is in decline. The theory is only an inference from the negative associations between intelligence and fertility, which the preponderance of the evidence indicates have been present in the United States, Britain, Greece and Sweden and probably other economically developed nations, and further supported by the negative associations between intelligence and number of siblings. (pp. 110–11)

One might possibly argue that the genetic decline and the environmental rise is somewhat interrelated, since modern societies enable more people to thrive and survive through various welfare services, improved medication in particular, despite being born into a lower-SES family and environment (Lynn 1996). Nonetheless, the decrease of infant mortality rates among the poor occurred earlier than the environmental processes that magnified cognitive ability test scores (Flynn 2012; Lynn 1996; Lynn 2009; see also Flynn and Shayer 2018). However, it seems that currently the processes are happening simultaneously in many national contexts (Dutton et al. 2016) which thus signifies parallelization.

At this point, cognitive ability research serves as an instantiation of parallel processes which have a global magnitude but might take a different route in the future due to new techniques in neuroscience (Haier 2017) and/or some unforeseeable event that change the fertility patterns. Education is important, not least for the world’s poor (e.g., Psacharopoulos 2018; Clots-Figueras 2018), and regardless of the specific fertility patterns in developing countries one can be rather sanguine about the rapid boosts in cognitive ability and educational attainment that occur as soon some degree of global equality narrowing is identified. However, in order to have a more comprehensive picture one must account for these two patterns rather than use a singular approach.

## Is Parallelization Yet another Form of Dialectic?

Dialectics, whether Hegelian, Marxist, or post-Marxist (e.g., Laclau and Mouffe 2001), is to some extent clearly associated with all the three dimensions of Pieterse’s cultural globalization model. Homogenization is indirectly linked to dialectics, since such neo-liberal or even neo-imperialist processes of global economics impinging upon other localities might lead to reactions in the forms of the more benign hybridization or the malign polarization. For instance, Huntington (1993) stresses that the Islamic resurgence is, in part, an implication of Western arrogance. This may not explain why Saudi Arabia and Iran quarrel, but the revolution in Iran in the 1970s was nevertheless partly a response to Western – especially British and American – imposition (Tehranian 2003; Abrahamian 1993). Hybridization is always a form of “synthesis” of different physical or discursive elements and as such associated with dialectical processes, whereas polarization is a form of dialectics in the broader sense, sometimes with merely discursive but occasionally with physical consequences.

However, parallelization is different since the processes are associated, often interdependent but not necessarily “dialectic” in the precise sense. Furthermore, as Ratner and Liu (2003) stress, dialectics leads to new ideas whereas parallel processes constitute physical phenomena that rather are related to a particular topic. Rising cognitive ability scores, as measured by standardized IQ tests such as Raven’s Progressive Matrices, WAIS or WISC, do not require the fertility rates to be constituted in a particular way to occur – instead, it seems as if they rise in spite of some slight decline in upper-middle class fertility rates. It is just the case that they would likely rise even more, for a certain period, if genotypic decline does not occur. A researcher might study one of these processes but should be aware of both, sometimes as a form of Popperian falsification criteria (Lakatos 1976) and thus to test if a pattern really exists or if the evidence is cherry-picked in order to fit a particular line of argument. Parallelization means exactly the opposite, that two or more patterns co-exist, although one might be predominant (for example the Flynn effect is more dominant than the slow and small genotypical decline identified in some countries by Lynn and Harvey 2008).

Similarly, economic growth which leads to high rates of carbon emissions, in the past and foreseeable future, does not automatically lead to a response to the problem that it has caused or may lead to. It is just simply the case that economic growth, interestingly, is related to the facilitation of infrastructure and resilience against climate change-related collateral damage and natural disasters like earthquakes. There is no dialectic merging or conflict present in these processes, neither any necessity. Building resilience is simply a human response, especially locally, to the natural environment and climate change which hinges upon relative economic growth and wealth (i.e., Japan compared to the Philippines). This does not imply that parallel processes happen haphazardly but that they do not necessarily occur as “dialectic” phenomena.

Congruent patterns may be identified with regard to secularization versus the rise of religion and religious pluralism. Indeed, secularism at least is associated with increasing religious pluralism in for instance several Western countries, but the global patterns of increased secularization occur simultaneously as opposite courses (i.e., the resurgence of religions, such as Islam, Christianity, and New Age). For instance, it might very well be the case that the ethnic Chinese population in Malaysia is becoming more atheist-oriented, while the Muslim population is increasingly conforming to Sunni orthodoxy and orthopraxy (Moustafa 2014; Fulow 2012: Tong and Turner 2008). Nonetheless, it would be a mistake to not stress the interdependence of these processes and peoples (Riesebrodt 2014). That these individuals and groups interact and influence each other is likely, why the hybrid or proculturation potential is present (Pieterse 2015; Gamsakhurdia 2019). The main point is that the two major patterns, regardless of outcome at a later step, are parallel.

## Concluding Remarks

Throughout the text, I have stressed the continuing significance of earlier theories of cultural globalization, that often tend to complement rather than contradict each other, since the world is complex to the extent that a singular pattern is not prevalent. While Pieterse (2015) underlines hybridization as the most dominant trajectory in a larger view of globalization as a process intertwined with world history and world culture, it does not account for other instances which lean more towards parallelization, that is, the multidirectional processes of psychological, semiotic (Valsiner 2014) and physical phenomena. By providing four exemplifications of parallelization, I have highlighted instances where at least two main processes related to a particular topic or phenomenon occur more or less simultaneously. For instance, as I write this, carbon emissions take place at astonishingly high rates through the technology and trade linked to global capitalism, while the economic growth builds more resilience in terms of infrastructure and ability to handle the costs. New influxes of migrants are entering for instance the US or Sweden, although at a somewhat slower pace than earlier phases due to national-populist agendas, which pose a risk of further segregation and inequality. However, at the same a large share of individuals from the same groups are struggling to accommodate to the host society and attain about the same education and earnings as the majority population.

Since several of the highlighted examples transcend cultural globalization even in the broader sense, the application of parallelization in the social sciences might be quite extensive. Thus, further research may look both wider and deeper into these and other topics and processes connected to parallelization, such as the relationship between macro cultural variables and psychology (e.g., Ratner 2012; Morselli 2013). Moreover, as some patterns, especially migration, segregation, and integration, might be interdependent, parallelization can be discussed as more intertwined processes (e.g., Valsiner 2014) and include more than just two major patterns.

## References

[CR1] Abrahamian E (1993). Khomeinism. Essays on the Islamic Republic.

[CR2] Alesina A, Devleeschauwer A, Easterly W, Kurlat S, Wacziarg R (2003). Fractionalization. Journal of Economic Growth.

[CR3] Baker D (2008). Korean spirituality.

[CR4] Baker, D. (2011). Globalization, nationalism, and Korean religion in the 21^st^ century. *Asia Pacific Perspectives,* X (1).

[CR5] Berger, P. (1999). Berger (ed.), *The desecularization of the world: resurgent religion and world politics*. Grand Rapids: William B. Erdmans publishing company.

[CR6] Bhabha H (1994). The location of culture.

[CR7] Borghans L, Golsteyun B, Heckman J, Humphries J (2011). Identification problems in personality psychology. Personality and Individual Differences.

[CR8] Cai Y, Lontzek TS (2019). The social cost of carbon with economic and climate risks. Journal of Political Economy.

[CR9] Casanova J (1994). Public religions in the modern world.

[CR10] Casanova J (2007). Re-thinking secularization: A global comparative perspective. Religion, Globalization and Culture (pp. 101**–**120).

[CR11] Cavalli-Sforza LL (2001). Genes, people, and languages.

[CR12] Ceci S, Williams W (2013). A qualitative synthesis of the Flynn effect. Measurement: Interdisciplinary Research and Perspectives.

[CR13] Ceci S (1991). How much does schooling influence general intelligence and its cognitive components? A reassessment of the evidence. Developmental Psychology.

[CR14] Clarke L, Edmonds J, Krey V, Richels R, Rose S, Tavoni M (2009). International climate policy architectures: Overview of the EMF 22 international scenarios. Energy Economics.

[CR15] Clots-Figueras I, Lomborg B (2018). Benefits and costs of the gender equality targets for the Post-2015 development agenda. Prioritizing development. A cost benefit analysis of the united Nation’s development goals.

[CR16] Dutton E, van der Linden D, Lynn R (2016). The negative Flynn effect: A systematic literature review. Intelligence.

[CR17] Ekberg J (1999). Immigration and the public sector: Income effects for the native population in Sweden. Journal of Population Economics.

[CR18] Engelhardt, L. Church, J. Harden, P. Tucker-Drob, E. (2018). Accounting for the shared environment in cognitive abilities and academic achievement with measured socioecological contexts. Developmental Science, 22 (1). 10.1111/desc.12699 PMC6294670 30113118

[CR19] Fairclough N (1992). Intertextuality in critical analysis. Linguistics and Education.

[CR20] Ferguson N (2011). Civilization. The west and the rest.

[CR21] Flynn J (2012). Are we getting smarter? Rising IQ in the twenty-first century.

[CR22] Flynn J, Shayer M (2018). IQ decline and Piaget: Does the rot start at the top?. Intelligence.

[CR23] Fulow, C. (2012). Secularism in Malaysia, in *Making sense of the secular. Critical Perspectives from Europe to Asia*, Ghosh, R. (ed.) (pp. 208–218). New York & London: Routledge.

[CR24] Galiana I, Lomborg B (2018). Benefits and costs of the climate change targets for the post-2015 development agenda. Prioritizing development: A cost benefit analysis of the United Nations’ sustainable development goals.

[CR25] Gamsakhurdia VL (2020). The origins and perspectives of ‘culture’ – Is it relevant anymore?. Human Arenas.

[CR26] Gamsakhurdia VL (2019). Proculturation: Self-reconstruction by making “fusion cocktails” of alien and familiar meanings. Culture & Psychology.

[CR27] Geels FW (2014). Regime-resistance against low-carbon transitions: Introducing politics and power into the multi-level perspective. Theory, Culture & Society.

[CR28] Gentile M (2015). West oriented in the east-oriented Donbas: A political stratigraphy of geopolitical identity in Luhansk, Ukraine. Post-Soviet Affairs.

[CR29] Gorski, P., Kim, DK., Torpey, J., VanAntwerpen, J. (2012). The post-secular in question*. Religion in contemporary society*. New York: New York University Press.

[CR30] Gorski P, Ates A (2008). After secularization?. Annual Review of Sociology.

[CR31] Gärdqvist, A. (2006). Stora utbildningsskillnader mellan invandrargrupper. Välfärd, 4. Available at https://www.scb.se/statistik/_publikationer/BE0801_2006K04_TI_02_A05ST0604.pdf.

[CR32] Greenfeld KT (1994). Speed tribes: Children of the Japanese bubble.

[CR33] Harlow E, Berg E, Barry J (2013). Neoliberalism, managerialism and the reconfiguring of social work in Sweden and the United Kingdom. Organization.

[CR34] Harvey D (2007). A brief history of neoliberalism.

[CR35] Heelas P (1996). The new age movement: The Celebration of the self and the sacralization of modernity.

[CR36] Hernæs Ø, Markussen S, Røed K (2019). Television, cognitive ability, and high school completion. Journal of Human Resources.

[CR37] Haier R (2017). The neuroscience of intelligence.

[CR38] Hannerz U (1992). Cultural Complexity.

[CR39] Huntington S (1996). The clash of civilizations and the remaking of world order.

[CR40] Huntington S (1993). The clash of civilizations?. Foreign Affairs.

[CR41] Inoue, N. (2007). Globalization and religion: The cases of Japan and Korea, in Beyer, Peter. Beaman, Lori G (ed.), *Religion, Globalization, and Culture*. Leiden: Brill: 453–472.

[CR42] Jahoda G (2012). Critical reflections on some recent definitions of “culture”. Culture & Psychology.

[CR43] Jin DJ, Ryoo W (2014). Critical interpretation of hybrid K-pop: The global-local paradigm of mixing English mixing in lyrics. Popular Music and Society.

[CR44] Jonsson G (2015). Can the Japan-Korea dispute on “comfort women” be resolved?. Korea Observer.

[CR45] Kolk, M. Barclay, K. (2019). Cognitive ability and fertility among Swedish men born 1951–1967. Evidence from military conscript registers. Proceedings of the Royal Society B: Biological Science, 286 (1902). 10.1098/rspb.2019.0359 PMC6532519 31064299

[CR46] Laclau, E., & Mouffe, C. (2001). *Hegemony and socialist strategy. Towards a radical democratic politics* (second ed.). London: Verso.

[CR47] Lakatos I (1976). Falsification and the methodology of scientific Programmes.

[CR48] Lee J, Zhu M (2015). The Asian American achievement paradox.

[CR49] Lie J (2014). K-pop: Popular Music, Cultural Amnesia, and Economic Innovation in South Korea.

[CR50] Lynn R, Harvey J (2008). The decline of the world’s IQ. Intelligence.

[CR51] Lynn R (2009). What has caused the Flynn effect? Secular increases in the development quotient of infants. Intelligence.

[CR52] Lynn R (1996). Dysgenics. Genetic deterioration in modern populations.

[CR53] Malmqvist K (2015). Satire, racist humor and the power of (un)laughter: On the restrained nature of Swedish online racist discourse targeting EU-migrants begging for money. Discourse & Society.

[CR54] Martin D (2005). On secularization: Towards a revised general theory.

[CR55] Martin D (1978). A general theory secularization: Towards a revised general theory.

[CR56] Moghaddam FM (2009). Commentary: Omniculturalism: Policy solutions to religious fundamentalism in the era of fractured globalization. Culture & Psychology.

[CR57] Mouffe C (2013). Agonistics. Thinking the world politically.

[CR58] Mouffe C (2005). The return of the political.

[CR59] Mouffe C (1999). The challenge of Carl Schmitt.

[CR60] Morselli D (2013). The olive tree effect: Future time perspective when the future is uncertain. Culture & Psychology.

[CR61] Moustafa T (2014). Judging in God’s name. State power, secularism, and the politics of Islamic law in Malaysia. Oxford Journal of Law and Religion.

[CR62] Norris P, Inglehart R (2012). Sacred and secular: Religion and politics worldwide.

[CR63] Nisbett R, Aronson J, Blair C, Dickens W, Flynn J, Halpern D (2012). Intelligence: New findings and theoretical developments. American Psychologist.

[CR64] Nisbett R (2010). Intelligence and how to get it: Why schools and cultures count.

[CR65] Nordhaus W (2015). The climate casino. Risk, uncertainty and economics for a warming world.

[CR66] Nyheter idag. (2019). SD går om S – här är alla överraskningar i Sentios augustimätning. Available at https://nyheteridag.se/plus/sd-gar-om-s-har-ar-alla-overraskningar-i-sentios-augustimatning/.

[CR67] Pieterse JN (2015). Globalization and culture: Global Mélange.

[CR68] Pieterse, J. N. (1996). Globalisation and culture: Three paradigms. *Economic and Political Weekly, 31*(23), 1389–1393.

[CR69] Pieterse JN (1994). Globalisation as hybridisation. International Sociology.

[CR70] Pietschnig J, Voracek M (2015). One century of global IQ gains: A formal meta-analysis of the Flynn-effect. Perspectives on Psychological Science.

[CR71] Plomin R (2018). Blueprint: How DNA makes us who we are.

[CR72] Psacharopoulos G, Lomborg B (2018). Education targets for the Post-2015 development agenda. Prioritizing development: A cost benefit analysis of the United Nations’ sustainable development goals.

[CR73] Ratner C (2012). Macro cultural psychology: A political philosophy of the mind.

[CR74] Ratner C, Liu L (2003). Theoretical and methodological problems in cross-cultural psychology. Journal for the Theory of Social Behavior.

[CR75] Ribberink E, Achterberg P, Houtman D (2018). Religious polarization: contesting religion in secularized Western European countries. Journal of Contemporary Religion.

[CR76] Riesebrodt, M. (2014). Religion in the modern world: Between secularization and resurgence. European University Institute, Italy: Max Weber lecture series, no. 2014/01.

[CR77] Riesebrodt M (2010). The promise of salvation. A theory of religion.

[CR78] Rindermann H (2018). Cognitive capitalism. Human capital and the wellbeing of nations.

[CR79] Ritzer G (1993). The McDonaldisation of society. Pine/Forge Sage.

[CR80] Rodrik D (2011). The globalization paradox. Democracy and the future of world politics.

[CR81] Ritchie S, Tucker-Drob E (2018). How much does education improve intelligence?. Psychological Science.

[CR82] Sanandaji T (2018). Massutmaning.

[CR83] Schieber B, Vishkin U (1988). On finding lowest common ancestors: Simplification and parallelization. SIAM Journal on Computing.

[CR84] Schmitt, C. (2009). [1932]. Der Begriff des Politischen. Berlin: Duncker & Humblot.

[CR85] Schwartz S (2019). Measuring vulnerability and deferring responsibility: Quantifying the Anthropocene. Theory, Culture & Society.

[CR86] Stark R (1999). Secularization R.I.P.. Sociology of Religion.

[CR87] Symaco LP (2013). Geographies of social exclusion: Education access in the Philippines. Comparative Education.

[CR88] Tehranian M, Saikal A, Schnabel A (2003). Disenchanted worlds: Secularization and democratization in Middle East. Democratization in the Middle East: Experiences, struggles and challenges.

[CR89] Tong JK-C, Turner BS (2008). Women, piety and practice: A study of women and religious practice. Contemporary Islam.

[CR90] Turkheimer E, Haley A, Waldron M, D’Onofrio B, Gottesman I (2003). Socioeconomic status modifies heritability of IQ in young children. Psychological Science.

[CR91] Valsiner J (2014). An invitation to cultural psychology.

[CR92] Vogiazides L, Mondani H (2020). A geographical path to integration? Exploring the interplay between regional context and labour market integration among refugees in Sweden. Journal of Ethnic and Migration Studies.

[CR93] World Values Survey. (2019). Available at http://www.worldvaluessurvey.org/WVSContents.jsp.

[CR94] Yasumura, S. (2014). Evacuation effect on excess mortality among institutionalized elderly after the Fukushima Daiichi nuclear power plant accident. *Fukushima Journal of Medical Science*, 60. 10.5387/fms.2014-13 25283975

[CR95] Yoon K (2017). Global imagination of K-pop: Pop music fans’ lived experiences of cultural hybridity. Popular Music and Society.

[CR96] Zhang Z (2011). Assessing China’s carbon intensity pledge for 2020: Stringency and credibility issues and their implications. Environmental Economics and Policy Studies.

